# Supplementary material for: Carbamazepine for Chronic Muscle Pain: A Retrospective Assessment of Indications, Side Effects, and Treatment Response
Source: Brain Sci. 2023 Jan 10;13(1):123. doi: 10.3390/brainsci13010123 (PMC9857021; doi:10.3390/brainsci13010123)
Supplement: Supplementary file 1 [file brainsci-13-00123-s001.zip › brainsci-2088819-supplementary.docx]

##### **Carbamazepine for chronic muscle pain: a retrospective assessment of indications, side-effects and treatment response**

Tabea M. Dyong (MD)^1^, Burkhard Gess (MD)^1^, Christina Dumke (MD)^1^, Roman Rolke (MD)^2^, Maike F. Dohrn (MD)^1,3^

1 Department of Neurology, Medical Faculty RWTH Aachen University, 52074 Aachen, Germany

2 Department of Palliative Medicine, Medical Faculty RWTH Aachen University, 52074 Aachen, Germany

3 Dr. John T. Macdonald Foundation, Department of Human Genetics and John P. Hussman Institute for Human Genomics, University of Miami, Miller School of Medicine, 33136 Miami, Florida, USA

**Supplementary material**

Methods S1: Detailed neurological assessment

We performed a detailed neurological examination in all patients, including gait, deep tendon reflexes, and muscle strength according to the modified Medical Research Council (MRC) rating scale(37). To obtain the sensory status, we assessed the perception of light touch, pinprick, temperature, position, and vibration on the upper and lower extremities, and documented the respective degree of abnormality, if applicable.

Table S1: patient overview and demographics

|  | diagnosis | | MUO | MET | MIT | MYO | NM | MCTB | GSD5 | CCD | PMC | FSHD1 | CNM | ANM | PROMM | NRM | MOS |
| --- | --- | --- | --- | --- | --- | --- | --- | --- | --- | --- | --- | --- | --- | --- | --- | --- | --- |
| sex | m:f  (age) | | 9:7  (49.5:47.4) | 1:0  (56) | 3:0  (45.3) | 2:0  (58.5) | 18:0  (48.72) | 1:0  (71) | 0:1  (25) | 1:1  (45:61) | 2:0  (48) | 1:1  (80:65) | 1:1  (43:60) | 0:1  (51) | 1:0  (54) | 1:0  (46) | 1:0  (54) |
| general  information | aoo | | 33.19 | 20 | 46 | 53 | 39.44 | 58 | 7 | 25 | 19.5 | 52 | 44 | 45 | 27 | 10 | ND |
|  | duration | | 15.45 | 36 | 5 | 5.5 | 9.28 | 13 | 18 | 28 | 28 | 13 | 12.5 | 4 | 27 | 35 | ND |
| first  symptoms | myalgia | | 16 | 1 | 3 | 2 | 14 | 1 | 1 | 1 | 2 |  | 2 | 1 | 1 | 1 | ND |
|  | muscle weakness | | 3 |  | 2 |  | 3 | 1 |  |  |  | 2 |  |  |  | 1 | ND |
|  | muscle cramps | | 5 |  |  | 1 | 11 |  | 1 |  | 1 |  | 1 | 1 | 1 |  | ND |
|  | fasciculations | | 3 |  |  |  | 12 |  |  |  |  |  |  |  |  |  | ND |
|  | muscle stiffness | | 1 |  |  |  | 1 |  |  |  | 2 |  |  | 1 |  |  | ND |
|  | rhabdomyolysis | | 1 |  |  |  |  |  |  |  |  |  |  |  |  |  | ND |
|  | elevated CK levels | | 1 |  |  | 1 |  |  |  | 1 |  |  | 1 |  |  |  | ND |
|  | arthralgia | |  |  |  |  | 1 |  |  |  |  |  |  |  |  |  | ND |
|  | recurring shoulder luxations | |  |  |  |  |  |  |  | 1 |  |  |  |  |  |  | ND |
| current  symptoms | myalgia | | 16 | 1 | 3 | 2 | 14 | 1 | 1 | 1 | 2 |  | 2 | 1 | 1 | 1 | ND |
|  | muscle weakness | | 6 |  | 2 |  | 4 |  |  | 1 | 1 | 2 | 1 |  | 1 |  | ND |
|  | muscle cramps | | 6 |  |  |  | 9 | 1 | 1 | 1 | 1 |  |  | 1 |  |  | ND |
|  | fasciculation | | 3 |  |  |  | 8 |  |  |  |  |  |  |  |  |  | ND |
|  | muscle stiffness | | 2 |  |  |  |  |  |  |  | 1 |  |  |  |  |  | ND |
|  | elevated CK levels | |  |  |  |  |  |  |  |  |  |  | 1 |  |  |  | ND |
|  | hypermobile joints | | 1 |  |  |  |  |  |  |  |  |  |  |  |  |  | ND |
| signs of  myopathy | unsteady gait | | 5 |  |  |  |  | 1 |  | 1 | 1 | 2 |  | 1 |  |  | ND |
|  | Gower’s sign | |  |  |  |  |  |  | 1 |  |  |  | 1 |  |  |  | ND |
|  | inability to squat | | 6 |  | 1 |  | 3 | 1 |  |  |  |  |  |  |  |  | ND |
|  | muscle atrophies | | 4 |  |  |  |  | 1 |  |  |  | 2 |  | 1 | 1 | 1 | ND |
|  | scapular winging | | 4 |  |  |  |  |  |  |  |  | 2 |  |  |  |  | ND |
|  | scoliosis | | 4 |  |  |  | 1 |  |  |  |  |  | 1 |  |  |  | ND |
|  | fasciculation | |  |  |  | 1 | 3 |  |  |  |  |  |  |  |  |  | ND |
|  | myotonia | |  |  |  | 1 | 2 | 1 |  |  |  |  |  |  |  |  | ND |
|  | myoclonus | |  |  |  | 1 |  |  |  |  |  |  |  |  |  |  | ND |
|  | rippling | |  | 1 |  |  |  |  |  |  |  |  |  |  |  |  | ND |
|  | diagnosis | | MUO | MET | MIT | MYO | NM | MCTB | GSD5 | CCD | PMC | FSHD1 | CNM | ANM | PROMM | NRM | MOS |
| signs of myopathy | weakness | |  |  |  | 1 |  |  |  |  |  |  |  |  |  |  | ND |
|  | ptosis | | 1 |  |  |  | 1 |  |  |  |  | 1 |  |  |  |  | ND |
|  | signe des cils | | 1 |  |  |  |  |  |  |  |  | 2 |  |  |  |  | ND |
| deep tendon  reflexes | normal | | 14 | 1 | 2 | 2 | 16 |  | 1 | 2 | 2 |  | 2 | 1 | 1 | 1 |  |
|  | reduced | | 2 |  | 1 |  | 2 | 1 |  |  |  | 2 |  |  |  | 1 | 1 |
| MRC  [x/5] | arm  abduction | 5 | 16 |  | 2 | 2 | 17 |  | 1 | 2 | 2 | 1 | 2 | 1 | 1 | 1 | 1 |
|  |  | 4 |  | 1 | 1 |  | 1 | 1 |  |  |  |  |  |  |  |  |  |
|  |  | 3 |  |  |  |  |  |  |  |  |  |  |  |  |  |  |  |
|  |  | 2 |  |  |  |  |  |  |  |  |  | 1 |  |  |  |  |  |
|  | arm  extension | 5 | 16 |  | 1 | 2 | 17 |  |  | 2 | 2 | 2 | 2 | 1 | 1 | 1 | 1 |
|  |  | 4 |  | 1 | 1 |  | 1 |  | 1 |  |  |  |  |  |  |  |  |
|  |  | 3 |  |  |  |  |  | 1 |  |  |  |  |  |  |  |  |  |
|  |  | 2 |  |  | 1 |  |  |  |  |  |  |  |  |  |  |  |  |
|  | finger  spreading | 5 | 16 | 1 | 2 | 2 | 18 | 1 | 1 | 1 | 2 | 1 | 2 | 1 | 1 | 1 | 1 |
|  |  | 4 |  |  | 1 |  |  |  |  | 1 |  | 1 |  |  |  |  |  |
|  | hip  flexion | 5 | 8 |  | 1 | 1 | 16 |  |  | 1 | 2 |  | 1 |  |  | 1 | 1 |
|  |  | 4 | 8 | 1 | 2 | 1 | 2 | 1 |  |  |  | 2 |  | 1 | 1 |  |  |
|  |  | 3 |  |  |  |  |  |  | 1 | 1 |  |  | 1 |  |  |  |  |
|  | hip  extension | 5 | 11 |  | 1 | 2 | 17 |  | 1 | 1 | 2 |  | 2 | 1 |  | 1 | 1 |
|  |  | 4 | 5 | 1 | 2 |  | 1 | 1 |  | 1 |  | 2 |  |  | 1 |  |  |
|  | knee  flexion | 5 | 11 |  | 1 | 2 | 16 | 1 |  | 1 | 2 | 2 | 2 | 1 |  | 1 | 1 |
|  |  | 4 | 5 | 1 | 2 |  | 2 |  | 1 | 1 |  |  |  |  | 1 |  |  |
|  | knee  extension | 5 | 10 |  | 1 | 2 | 16 | 1 | 1 | 1 | 2 | 2 | 1 | 1 | 1 | 1 | 1 |
|  |  | 4 | 6 | 1 | 2 |  | 2 |  |  | 1 |  |  | 1 |  |  |  |  |
| NRS | before treatment | | 6.19 | ND | 10 | 9 | 6.58 | ND | 8 | 6 | 7 | ND | 6 | 8 | 5 | ND | 8 |
|  | under treatment | | 5 | ND | 9 | 2 | 3.5 | ND | 6 | 5 | 7 | ND | 6 | 7 | 1.5 | ND | 6 |
| carbamazepine | dosage (mean) | | 200 | 200 | 200 | 300 | 305.55 | 600 | 200 | 200 | 300 | 200 | 200 | 200 | 400 | 200 | 200 |
|  | duration (months) | | 4.55 | 5 | 27 | 19.5 | 16 | ND | 4 | 18 | 1 | 8 | 4 | 49 | 120 | ND | ND |
| painDETECT score | n | | 10 | 0 | 1 | 1 | 7 | 0 | 1 | 2 | 1 | 0 | 1 | 1 | 1 | 0 | 1 |
|  | current NRS [x/10] | | 4.4 | ND | 10 | 2 | 3.93 | ND | 9 | 6 | 7 | ND | 5.5 | 0 | 1.5 | ND | 7 |
|  | maximum NRS [x/10] | | 7.5 | ND | 10 | 2 | 6.79 | ND | 9 | 8.25 | 7 | ND | 9 | 8,5 | 7 | ND | 8 |
|  | diagnosis | | MUO | MET | MIT | MYO | NM | MCTB | GSD5 | CCD | PMC | FSHD1 | CNM | ANM | PROMM | NRM | MOS |
| painDETECT score | mean NRS [x/10] | | 4.7 | ND | 9 | 2 | 5.36 | ND | 7.5 | 6.5 | 7 | ND | 5.5 | 4.5 | 1.5 | ND | 6.5 |
|  | burning sensations [x/5] | | 1.2 | ND | 5 | 0 | 1.43 | ND | 0 | 2 | 0 | ND | 1 | 0 | 2 | ND | 4 |
|  | tingling or pricking sensations [x/5] | | 1.3 | ND | 4 | 3 | 2.71 | ND | 0 | 2 | 0 | ND | 2 | 0 | 0 | ND | 1 |
|  | light touch [x/5] | | 0.6 | ND | 3 | 0 | 0.14 | ND | 0 | 2 | 0 | ND | 0 | 0 | 0 | ND | 2 |
|  | pain attacks [x/5] | | 1.5 | ND | 4 | 3 | 1.57 | ND | 0 | 2 | 0 | ND | 1 | 2 | 0 | ND | 4 |
|  | cold or heat pain [x/5] | | 0.9 | ND | 1 | 0 | 0.86 | ND | 0 | 2 | 0 | ND | 0 | 0 | 0 | ND | 1 |
|  | numbness [x/5] | | 1.3 | ND | 4 | 3 | 2.14 | ND | 0 | 3 | 0 | ND | 0 | 0 | 0 | ND | 1 |
|  | light pressure [x/5] | | 1 | ND | 4 | 1 | 0.86 | ND | 0 | 0 | 0 | ND | 0 | 0 | 0 | ND | 5 |
|  | total points [x/35] | | 8.7 | ND | 25 | 10 | 10.43 | ND | 0 | 14 | 0 | ND | 4 | 2 | 2 | ND | 19 |
| QST | normal | | 7 | ND | ND | 1 | 3 | ND |  | 1 |  | ND |  | 1 | 1 | ND | ND |
|  | abnormal | | 3 | ND | ND |  | 2 | ND | 1 | 1 | 1 | ND | 1 |  |  | ND | ND |
| laboratory analyses | CRP [mg/l] | | 6.65 | ND | 3.25 | 3.15 | 1,46 | 1,3 | 14.4 | 4.35 | 4.23 | 1,65 | 3.85 | 0.38 | 1.1 | ND | 0.9 |
|  | CK [U/l] | | 132.65 | ND | 299.88 | 278.2 | 253.62 | 177,5 | 1391.33 | 490.33 | 136.67 | 290.5 | 987.5 | 818.75 | 2888 | 116 | 210 |
|  | AST [U/l] | | 26.08 | ND | 35 | 28.75 | 28.82 | 41,5 | 20 | 35 | 24.33 | 29.67 | 51 | 32.75 | 106 | 39 | 30 |
|  | ALT [U/l] | | 29.23 | ND | 34.6 | 29.75 | 32.58 | 75 | 23.33 | 49.5 | 25.33 | 24.67 | 46 | 46.25 | 117 | 50.5 | 12 |
|  | GGT [U/l] | | 33.7 | ND | 64.8 | 111.5 | 42.61 | 57 | 24.33 | 74.5 | 38.5 | 16.67 | 30 | 53.37 | 216 | 30 | 16 |
|  | vitamin B12 [pg/ml] | | 360.57 | ND | 333.33 | 160.67 | 483.18 | 314 | 266 | 815.5 | 562.67 | 316 | 541.67 | 358.5 | 360 | 713 | 420 |
|  | folic acid [ng/ml] | | 6.14 | ND | 4.7 | 4 | 9,66 | 5,2 | 7.9 | 7 | 8.13 | 6.1 | 4.8 | 5.45 | 11.9 | 18.8 | 10.1 |
|  | HbA1c [%] | | 5.57 | ND | 6.58 | 5.72 | 5.44 | 5,25 | 5.1 | 5.45 | 6.17 | 5.3 | 5.7 | 5.53 | 5.7 | 5.7 | 5.6 |
|  | CDT [%] | | 0.94 | ND | 1.3 | 1.3 | 1.13 | ND | ND | 1 | 1 | ND | 0.7 | 0.7 | 1.2 | ND | 1.1 |

Abbreviations: ALT = alanine transaminase; ANM = autoimmune necrotizing myopathy; AOO = age of onset; AST = aspartate aminotransferase; CCD = central core disease; CDT = carbohydrate-deficient transferrin; CI-SLE = carbamazepine-induced systemic lupus erythematosus; CK = creatine kinase; CNM = centronuclear myopathy; CRP = C-reactive protein; FSHD1 = facio-scapulo-humeral muscle dystrophy type 1; GGT = γ-glutamyl transpeptidase; GSD 5 = phosphorylase deficiency (McArdle disease); HbA1c = glycated hemoglobin; MCTB = myotonia congenita type Becker; MET = metabolic myopathy; MIT = mitochondrial myopathy; MOS = myositic overlap syndrome; MRC = Medical Research Council; MUO = myopathy of unknown origin; MYO = myofibrillar myopathy; ND = no data; NM = Neuromyotonia; NRM = nemaline rod myopathy; NRS = numeric rating scale; PMC = paramyotonia congenita; PROMM = proximal myotonic myopathy; QST = quantitative sensory testing

Results S1: Detailed laboratory analyses

We assessed a full hemogram, alanine transaminase (ALT), aspartate aminotransferase (AST), γ-glutamyl transpeptidase (GGT), creatinine, glomerular filtration rate (GFR), sodium, potassium, creatine kinase (CK), vitamin B12, folic acid, ferritin, glycated hemoglobin (HbA1c), thyroid stimulating hormone (TSH), carbohydrate-deficient transferrin (CDT), and C-reactive protein (CRP). For all parameters, we used the normative values of the RWTH Aachen university hospital.

The mean average CK level was 333.9$\pm$455.9 U/l (range= 45-2888 U/l, cut-off > 190 U/l (male) / 170 U/l (female), mean= 333.9$\pm$455.9 U/l, median= 186$\pm$455.9 U/l, n= 90). Forty-four CK values (49%, 34 male and 10 female) were below 170 U/l, one value (1%, male) was between 170 and 190U/l, and 45 values (50%, 34 male and 11 female) were higher than 190 U/l. The three highest values were 1054 U/l in a female patient with centronuclear myopathy, 2639U/l in a female patient with McArdle disease, and 2888U/l in one male patient with myotonic dystrophy type 2 (PROMM). Assessing potential side effects of carbamazepine treatment, we measured the enzymes AST, ALT (both also muscle enzymes), and GGT in serum. Nine AST values (10%, 2 male and 7 female) were elevated, (range= 19-106 U/l, cut-off > 50 U/l (male) / > 35 U/l (female), mean with medication= 31.68$\pm$19.38 U/l, mean without medication= 30.31$\pm$9,89 U/l, median with medication= 28$\pm$19.38 U/l, median without medication= 27$\pm$9.89 U/l, n= 87). Fourteen ALT values (18%, 8 male and 6 female) were elevated (range= 16-117 U/l, cut-off > 50 U/l (male) / > 35 U/l (female), mean with medication= 35.21$\pm$25 U/l, mean without medication= 33.3$\pm$18.13 U/l, median= with medication 29$\pm$25 U/l, median= without medication 26.5$\pm$18.13 U/l, n= 83). Sixteen GGT values (21%) were elevated (range= 12-216 U/l, cut-off > 60 U/l (male) / > 40 U/l (female), mean with medication= 76.53$\pm$51.67 U/l, mean without medication= 34.25$\pm$19.34 U/l, median with medication= 50$\pm$51.67 U/l, median without medication= 30$\pm$19.34 U/l, n= 77). These data do not show a significant elevation of AST (p= 0.805) or ALT (p= 0.9179) under carbamazepine, but GGT values were significantly higher (p= 0.0001) in patients treated with carbamazepine. Four patients (7%) reported that the liver enzymes had been elevated at external examinations requiring carbamazepine treatment to be stopped. However, we do not have access to the exact values. To rule out the potential confounder of pathological values that are typically associated with other origins of chronic pain, such as neuropathies, we measured vitamin B12, folic acid, CDT, and HbA1c. Two patients (5%) had vitamin B12 deficiency, and folic acid deficiency was seen in eight patients (19%). All patients with lower folic acid or vitamin B12 levels began substituting right afterwards. One patient (4%) had a higher CDT level (1.9%) than normal (< 1.5%) (n= 24). None of those patients showed signs of anemia or sensory deficits in the clinical examination. HbA1c levels (n= 43) were below 6.5% in 39 patients (91%). A slight elevation (6.7%) was observed in two and a significant elevation (7.7-7.9%) in one additional patient.

Abbreviations: ALT = alanine transaminase; AST = aspartate aminotransferase; CDT = carbohydrate-deficient transferrin; CK = creatine kinase; CRP = C-reactive protein; GFR = glomerular filtration rate; GGT = γ-glutamyl transpeptidase; HbA1c = glycated hemoglobin; PROMM = proximal myotonic myopathy; TSH = thyroid stimulating hormone

Table S2: QST original data

|  | CDT [°C] | WDT [°C] | TSL [°C] | PHS [n/3] | CPT [°C] | HPT [°C] | MDT [mN] | MPT [mN] | MPS | DMA | WUR | VDT [n/8] | PTT [kPa] |
| --- | --- | --- | --- | --- | --- | --- | --- | --- | --- | --- | --- | --- | --- |
| mean patients | -0.611 | -0.895 | -0.725 | 0.708 | 0.501 | -0.219 | 0.298 | 0.937 | 0.483 | -0.709 | 0.026 | -0.787 | -0.201 |
| SD patients | 1.061 | 0.896 | 0.781 | 1.197 | 1.091 | 1.074 | 1.073 | 1.082 | 2.122 | 0.581 | 1.263 | 1.449 | 1.113 |
| SEM patients | 0.217 | 0.183 | 0.159 | 0.244 | 0.223 | 0.219 | 0.219 | 0.221 | 0.433 | 0.119 | 0.258 | 0.296 | 0.232 |
|  |  |  |  |  |  |  |  |  |  |  |  |  |  |
| mean control | 0 | 5.273E-16 | -2.683E-16 | 0.125 | 8.326E-17 | -5.736E-16 | 1.099E-16 | -1.489E-15 | 6.476E-17 | -0.997 | -2.656E-16 | -2.821E-16 | -9.159E-16 |
| SD control | 1 | 1 | 1 | 0.612 | 1 | 1 | 1 | 1 | 1 | 0.016 | 1 | 1 | 1 |
|  |  |  |  |  |  |  |  |  |  |  |  |  |  |
| t-test | 0.046 | 0.002 | 0.007 | 0.039 | 0.104 | 0.467 | 0.325 | 0.003 | 0.319 | 0.019 | 0.936 | 0.337 | 0.517 |

Mean values of both groups including results for paired t-tests. We considered p-values < 0.05 significant. The values for cold detection threshold (CDT), warmth detection threshold (WDT), thermal linen sensations (TSL), mechanical pain threshold (MPT) and vibration detection threshold are significantly different in both groups.

Abbreviations: CDT = cold detection threshold; CPT = cold pain threshold; DMA = dynamic mechanical allodynia; HPT = heat pain threshold; MDT = mechanical detection threshold; MPS = mechanical pain sensitivity; MPT = mechanical pain threshold; PHS = paradoxical heat sensation; PPT = pressure pain threshold; SD= standard deviation; SEM= standard error of the mean; TSL = thermal sensory linen; VDT = vibration detection threshold; WDT = warm detection threshold; WUR = wind-up ratio

Supplementary Result S2: Pain reduction in male and female patients

| NRS before treatment (male) | NRS after treatment (male) | NRS difference  (male) | NRS before treatment (female) | NRS after treatment (female) | NRS difference  (female) |
| --- | --- | --- | --- | --- | --- |
| 9 | 2 | 7 | 8 | 6 | 2 |
| 7.5 | 4 | 3.5 | 4.5 | 3.5 | 1 |
| 7.5 | 6 | 1.5 | 8 | 6.5 | 1.5 |
| 10 | 8 | 2 | 8 | 8 | 0 |
| 5 | 1.5 | 3.5 | 3 | 3 | 0 |
| 6.5 | 3 | 3.5 | 6 | 6 | 0 |
| 4 | 6.5 | -2.5 | 5.5 | 5.5 | 0 |
| 5 | 2 | 3 |  |  |  |
| 7 | 2 | 5 |  |  |  |
| 6 | 4 | 2 |  |  |  |
| 10 | 9 | 1 |  |  |  |
| 6.5 | 3.5 | 3 |  |  |  |
| 7 | 7 | 0 |  |  |  |
| 5 | 5 | 0 |  |  |  |
| 8.5 | 3 | 5.5 |  |  |  |
| 6.5 | 5 | 1.5 |  |  |  |
| 8 | 6 | 2 |  |  |  |

We compared the change in NRS (numeric rating scale) scores in male (n= 17) and female (n= 7) patients separately. The NRS results before and after treatment were not significantly different (male to female before treatment: p= 0.3024, male to female after treatment: p= 0.335). However, the change in NRS scores was significantly different (p= 0.0245). From this retrospectively assessed real-life cohort, it is difficult to extrapolate a clinically relevant sex difference in pain reduction. Further studies with larger cohorts and a prospective, double-blind study design are needed to further explore this effect.
